# Medical News

**Published:** 1855-07

**Authors:** 


### MEDICAL NEWS.

Dr. L. S. Joynes has been appointed to the chair of Medical Jurisprudence and Physiology, in the Virginia Medical College, vacated by the resignation of Dr. Brown-Sèquard. Dr. A. E. Peticolas, has been elected to the chair of Anatomy, in the same Institution.

---

The Stethoscope having been sold by the Virginia State Medical Society, is now edited by Drs. Goodridge A. Wilson and Richmond A. Lewis.

Messrs. Parry & M'Millan will publish, in the course of a few days, a duodecimo volume, upon the "Mineral and Thermal Springs of the United States and Canada," by Dr. John Bell, of this city. It will be noticed in our next number.

---

The profession in Buffalo, learning that Dr. Austin Flint was about to sever his long connection with the Buffalo Medical Journal, and desirous of presenting him with some testimonial of their appreciation of his high character and endowments, have recently had a handsome engraving taken of him, for a copy of which we are indebted to the politeness of Dr. Sandford B. Hunt. As an evidence of the respect and admiration entertained for him by his professional associates, this farewell tribute must have been especially gratifying to Dr. Flint.

Dr. Hunt is now sole editor of the Journal.

---

In our account of the proceedings of the A. M. Association, Dr. Frank H. Hamilton, of New York, is made to say that it is impossible to unite a fractured femur without shortening. The following extract from the Buffalo Medical Journal, reports his language correctly :—

"Where, then, must we look for an answer to the question, Why are these prosecutions against surgeons so frequent? Let the gentlemen be assured, the causes are to be found in the *very imperfections of our art, and in our own unwillingness to admit these imperfections.* Surgeons have claimed too much, and it cannot certainly be expected that the world will demand of them less than they claim for themselves. Again and again surgeons have said that a fracture of femur might be generally made to unite without any shortening, while the fact is not so. Malgaigne, who is eminently an honest man, says, to make this bone unite in an adult person, where the fracture is sufficiently oblique to prevent the ends from supporting each other, is 'simply impossible,' (*simplement impossible.*)

"Let the profession be wiser in future, and acknowledge that they cannot perform impossibilities."

---

*Extracts from the Proceedings of the Ohio State Medical Society, at its meeting in Zanesville, June,* 1855.

AFTERNOON SESSION, JUNE 5th, 2 O'CLOCK, P. M.

Dr. Woodward in the chair.

Drs. Baker and Hills, in the absence of a previously appointed committee, were requested to conduct the president elect to the chair, which duty they performed. Upon taking the chair, Dr. Hempstead, in a few appropriate remarks, returned his thanks to the convention.

The order of the day was the valedictory address of Dr. Woodward. His theme was that of Ethics, generally, and the recent action of the

National Association, in particular. He presented this matter to the Society as officially presented to him by the Secretary of that Association.

In a beautifully written address, the Doctor dwelt at some length upon this action of the National body, and said that it would be much better for the profession if most of the restraints established by what is termed the Code of Ethics, were removed. These shackles are not imposed on other professions, and it would be found much better if each member was permitted to be governed by his own sense of right. It was strange that such a dignified body as the American Medical Association should indulge in such hasty action. It would have been far better to have appointed a committee to confer with the Ohio Association, when the matter could probably have been adjusted after due consideration. He recommended an adherence to the original resolutions, and such action as would express to that Association our sense of the indignity he considered they had offered us in their proceedings.

By the suggestion of Dr. Wright, who stated that other papers on the same subject would be presented to the convention, the valedictory of the Ex-President was laid upon the table for the present.

Dr. Wright, from a special committee, appointed at the last convention, to inquire into the expedieney of revising the Code of Medical Ethics, made a lengthy and very *ingenious* report, in which irony, sarcasm and ridicule were freely commingled with arguments, and all hurled with great force upon the devoted code. He deemed a few of its provisions very good, but common place; others were ridiculous; while some were arrogant and assuming. The code was formed, no doubt, with good intentions; but there was no necessity of any code. There was not enough for the good in it, and too much for the bad—who would use some of its provisions to enable them to carry on their evil designs. The great moral law was alone sufficient to guide every one who was fit to belong to the profession. He was desirous of action now. He did not believe in many rules. Some of the best societies have the fewest laws; while too much legislation has been the bane of others. He again expressed himself as decidedly in favor of the abolition of the code of ethics, and concluded the report with the following resolution:

*Resolved*, That the Ohio State Medical Society does not require the adoption of any code of ethics, as such, for kindness of intercourse, concert of action and scientific improvement—that the great moral code, declaring it our duty to do unto others as we wish others to do unto us, and our own sentiments as gentlemen, are sufficient for all proper professional and social intercourse.

The report and resolution were laid upon the table.

Upon a motion to make the report, together with the address of the ex-president, the special order for Wednesday morning at 9 o'clock, a somewhat lengthy and animated discussion took place, which was continued until the adjournment. It was finally carried.

Drs. Thompson, Baker, Lawson, Wright, Ball an others participated.

MORNING SESSION, JUNE 6th.

The report and resolutions of the special committee, appointed to inquire into the expediency of revising the code of ethics, together with the address of the ex-president, being the special order for this morning, were taken up, and after various motions, and a somewhat lengthy debate in regard to questions of order, a motion was finally adopted to bring the resolution accompanying the report, before the convention.

Prof. Baker took the floor, and addressed the House at some length, in opposition to the resolutions. He objected not only to the argument in the report of the committee, but the manner in which it was written and delivered. Still, he desired to treat the committee courteously. It was an easy matter to throw ridicule upon any subject by adopting a peculiar manner of treating it. But so far as the code was concerned, it had been read, re-read, and had been received with favor by the profession. It was sensible. A few trivial matters might have been left out, if all whom it was intended to reach were intelligent men, but this was not the case: and so far as he was concerned, the code was satisfactory. It was not expected to be compulsory upon the people; if any one did not show proper respect to his attending physician, it was not expected that his mouth could be shut, but we can claim that the patient should treat his physician courteously, and if he will not do so, he cannot expect the physician to attend upon him, and in a case of that kind, the latter would be sustained by the profession.

Prof. B. dwelt at some length upon the various articles of the code, and concluded by picturing the evil effects which would follow its abolition. It would bring the faculty down to a level with empirics, and destroy the dignity and usefulness of the profession.

In regard to the action of the State Society, at its last session, he thought it to be wrong, and the sooner it retraced its steps the better. He conceived the code to be the Bible of the profession in Cincinnati, and would be extremely sorry to see it mutilated.

On motion of Dr. Wm. Mussey, speeches were limited to ten minutes each, and no one had a right to occupy the floor twice, except by way of explanation.

A motion for the previous question was made, but afterwards withdrawn, and a motion for the indefinite postponement of the whole matter was lost.

The previous question was again called for, but subsequently the motion was withdrawn.

Dr. Murphy said he did not arise to discuss the subject of ethics. That was unnecessary. He considered that the question before the convention was, whether the Society should have an existence after this meeting had adjourned. He was satisfied, after conversation with many members of the profession in various parts of the State, and especially with young men, that, if the action of the last session is not rescinded, a new society will be formed. If he had been a member of the late convention of the National Medical Association, he would have gone farther than it did. He would have made the delegates from this State get down on their knees, and promise to rescind their action, and had it not been for the prudence and caution of our friends, this would have been done by the convention, instead of making its action prospective. The delegates should not have been allowed to take seats.

He said that he did not make these remarks in the way of a threat, but simply to say what would take place if this resolution was passed.

Dr. Hills wished to remark that he was a member of the National Convention, and that their action in regard to this matter was not hasty, as stated, but was taken after full consideration; that as first presented to that body it was with misunderstandings; that he took occasion to correct them, which modified the feelings, and hence the action was made prospective instead of immediate. He cared not to reply to the reflections in the report on the conduct of the representatives—the question now was on the abrogation of the entire code of ethics, and he felt that such an act would be disastrous to the Society—would practically dismember it, and be ruinous to the interests of the profession, generally.

Dr. R. Thompson made a few remarks in favor of the resolution. He was of the opinion that any one should have a right to his own invention.

After he concluded, Dr. ——— called for the previous question, but it was withdrawn for the purpose of giving the chairman of the special committee an opportunity to close the debate.

Dr. Wright then took the floor, and briefly replied to some of the objections urged against the resolution. He said he did not care how soon this Society would be severed from the American Association, if a connection with that body could only be held by being subservient to it. He considered the Society in Ohio superior to it, and at some time it might happen that they would be glad of our assistance.

So far as the language of the report and the manner of delivery was concerned, that had nothing to do with its statements or arguments. They were true or false, and his tone could not change their effect.

Men, without the code, would be governed by principle; now, the code governs them. For his part, he would rather trust to the honor of a gentleman than the regulations of the code. He, therefore, asked for its abolition, and if any location desired rules or regulations, they could frame a code adapted to their peculiar position. He regarded the system of ethics as mere twaddle, and was unwilling that the Ohio State Medical Society should humble itself and rescind its former action in order to gain favor with the American Medical Association.

The motion to adopt the report and accompanying resolutions of the committee was then lost by a very large majority.

The following resolutions offered immediately, by Dr. T. W. Gordon, were adopted almost unanimously, without debate.

*Resolved*, That the resolution offered by Dr. Grant, (a member of this Society, and not at this, or at that time, a practitioner of medicine,) at the last session of this Society, which says "that it is not derogatory to medical dignity, or inconsistent with medical honor, for medical gentlemen to take out a patent right for surgical or med[illegible] that the [illegible]nts," was offered at a time when many members of the S[illegible] or adaptation for their homes, and was not, therefore, the sense of this Society.

*Resolved, also*, That said resolution is in direct opposition to the code of Medical Ethics adopted by the Society, and therefore be it further

*Resolved*, That said resolution offered by Dr. Grant, and adopted by this Society be, and is hereby rescinded.

On motion of Dr. Murphy, the Secretary was instructed to forward to the Secretary of the American Association and to the Executive committee at Detroit, a statement of the action of this convention, and a copy of the resolutions rescinding those of last year.—*Medical Counsellor*, (*Columbus, Ohio.*)

